# Predicting Reading and Spelling Disorders: A 4-Year Prospective Cohort Study

**DOI:** 10.3389/fpsyg.2016.00337

**Published:** 2016-03-09

**Authors:** Lucia Bigozzi, Christian Tarchi, Corrado Caudek, Giuliana Pinto

**Affiliations:** ^1^Department of Education and Psychology, University of FlorenceFlorence, Italy; ^2^Department of Neurosciences, Psychology, Drug Research and Child Health, University of FlorenceFlorence, Italy

**Keywords:** reading disorder, spelling disorder, predictors, phonological awareness, invented spelling, textual competence

## Abstract

In this 4-year prospective cohort study, children with a reading and spelling disorder, children with a spelling impairment, and children without a reading and/or spelling disorder (control group) in a transparent orthography were identified in third grade, and their emergent literacy performances in kindergarten compared retrospectively. Six hundred and forty-two Italian children participated. This cohort was followed from the last year of kindergarten to third grade. In kindergarten, the children were assessed in phonological awareness, conceptual knowledge of writing systems and textual competence. In third grade, 18 children with a reading and spelling impairment and 13 children with a spelling impairment were identified. Overall, conceptual knowledge of the writing system was the only statistically significant predictor of the clinical samples. No differences were found between the two clinical samples.

## Introduction

Spelling disorders have often been found to be associated with reading disorders (Lyon et al., 2003), a finding that is further supported by the consideration that reading and spelling performances are also associated in the general population (Bates et al., 2006). The existence of associations between disorders poses questions about whether they share the same cognitive basis (Pennington, 2006). Furthermore, studies on reading and spelling disorders need to take the level of consistency of the mapping between letters and sounds in words into account as a level of explanation, and increase our understanding of transparent orthographies (Ziegler et al., 2010). This 4-year prospective cohort study compared, in kindergarten, the early cognitive skills of a sample of spelling-disabled pupils (SD) with those of a sample of reading-and-spelling-disabled pupils (RSD) and with those of a sample of children without a reading and/or spelling disorder (control group). The study was conducted in an Italian-speaking population and is characterized by the fact that Italian provides a transparent orthography. A better knowledge of the differences in the early cognitive skills between these three groups of children can contribute to identifying the predictors of spelling impairments, which are still underspecified and poorly understood (American Psychology Association, 2013).

### Definition of Reading and Spelling Disorders

In line with the findings suggesting an association between learning disorders — e.g., between reading (dyslexia) and spelling disorders (dysorthographia; Lyon et al., 2003; Egan and Tainturier, 2011; Moll et al., 2014) — the latest edition of the American Psychiatric Association’s Diagnostic and Statistical Manual of Mental Disorders (DSM-5) combines the DSM-IV diagnoses of a number of disorders: reading disorder, mathematics disorder, disorder of written expression, and learning disorder not otherwise specified (American Psychology Association, 2013). The DSM-5, however, stresses the possibility of a dissociation between these different learning disorders (Berninger et al., 2015), as it requires separate coding of deficits belonging to specific domains. Thus, dyslexia is defined as a learning disorder that produces an impairment in reading and requires the specification of whether word reading accuracy, reading rate or fluency, spelling, or reading comprehension are compromised (ICD-9 code: 315.00; ICD-10 code: F81.0). Likewise, dysorthographia is defined as a learning disorder with an impairment in written expression, and it requires the specification of whether spelling accuracy, grammar and punctuation accuracy, clarity, or organization of written expression are compromised (ICD-9 code: 315.2; ICD-10 code: F81.81). Following the indication of the DSM-5 (American Psychology Association, 2013), in this study, we identified two clinical groups: (1) children with a specific learning disorder with an impairment in reading accuracy and fluency (315.00), which was associated with a specific learning disorder with an impairment in written expression, in particular in spelling accuracy (315.2), and (2) children with a specific learning disorder with an impairment in written expression only, in particular in spelling accuracy (315.2). These disorders were diagnosed in absence of comorbidity with other neuro-developmental (e.g., ADHD) or mental disorders (e.g., anxiety disorder) that typically co-occur with specific learning disorders.

### Spelling in Reading and Writing

Interestingly, the term “spelling” is used for both reading and writing. Whereas the use of spelling disorder for a writing disorder is quite obvious, many influential definitions of the reading disorder also include spelling problems in children (Lyon et al., 2003; Pennington, 2009), as well as in adults (Afonso et al., 2015). For example, according to the International Dyslexia Association and National Institutes of Child Health and Human Development, a reading disorder is characterized by difficulties with accurate and/or fluent word recognition and by poor spelling and decoding abilities.

By focusing on the spelling impairment, this study’s overall aim is to contribute to a better understanding of the association between reading and spelling disorders. In fact, spelling is a bridging skill between reading and writing which, if impaired, produces a reading-writing disorder. However, spelling is asymmetrical, as it is more difficult when writing than when reading. Thus, a mild spelling impairment may allow pupils to master the easier process (i.e., reading), but not the more difficult one (i.e., writing). Conversely, a severe spelling impairment may cause pupils to struggle in both processes, reading and writing. According to past research, a specific writing impairment might be a residual problem of those pupils who have managed to compensate for earlier reading difficulties (Newman et al., 1993). Studies on spelling disorders vs. reading-spelling disorders are lacking, mostly because research on reading disorder has focused on reading only, thus neglecting its relation with spelling disorders (Morken and Helland, 2013).

### The Role of the Transparency of the Writing System

Reading and spelling disorders change depending on the level of transparency of a writing system (i.e., how consistently letters map onto sounds —Paulesu et al., 2001; Raman and Weekes, 2005). In transparent writing systems (e.g., Italian or German), in which each letter is almost always pronounced in the same way in different words, the typical problem of children with a reading disorder is reading fluently, rather than accuracy (Barca et al., 2006; Zoccolotti et al., 2014, 2015). Conversely, in opaque writing systems (e.g., English or French), in which some letters are pronounced in different ways in different words, children with a reading disorder struggle to read fluently and also correctly (Wimmer and Mayringer, 2002; Wimmer and Schurz, 2010). Instead, children with a spelling impairment are inaccurate writers in both orthography systems, transparent and opaque (Angelelli et al., 2010). It should also be noted that, in most languages, spelling is more difficult than reading (Newman et al., 1993). This difficulty gap is enhanced in transparent orthographies, in which the regularity of the orthographic system is higher in grapheme–phoneme relations (forward regularity) than in phoneme–grapheme relations (backward regularity; Wimmer and Mayringer, 2002; Notarnicola et al., 2012) — for example, in Italian the phoneme /k/ can correspond to two different graphemes, ‘c’ as in /kwͻko/ (‘cuoco,’ en. tr. ‘chef’), or ‘q’ as in /kwì/ (‘qui,’ en. tr. ‘here’).

The Italian language, because of its characteristics of transparency and reading-spelling asymmetry, provides optimal conditions to study spelling impairment as an independent disorder, and spelling impairment in association with a reading impairment. In addition, Italian spelling in writing plays a leading role for the acquisition of both, reading and writing (Pinto et al., 2015), which makes the exploration of the early predictors of this process even more crucial.

### Predictors of Reading and Spelling Disorders

In this study, children with a spelling disorder (SD), children with a reading and spelling disorder (RSD), and children without a reading/spelling disorder (control group) were identified in third grade. Their emergent literacy performances in the last year of kindergarten were then retrospectively compared. According to Pennington (2006), in fact, finding a common antecedent deficit would confirm the severity hypothesis, according to which RSD is an earlier and more severe form of the same etiology underlying the SD. In this paragraph, we discuss the literature on the predictors of reading and spelling disorders.

Although spelling has not received a similar amount of research interest as reading, there are several studies available on predictors of spelling, also in transparent orthographies. Many of these studies support the existence of different cognitive predictors of reading and spelling. According to Vaessen and Blomert (2013), among the most important predictors of reading, only phonological awareness (i.e., the ability to identify and manipulate units of sounds) and letter-sound matching skills (i.e., the ability to match letters to corresponding speech sounds) are also predictors of spelling, especially in transparent orthographies.

Among the aforementioned skills, phonological awareness is the most debated, in particular concerning its relationship with the acquisition of reading and spelling skills across different languages. For quite some time, phonological awareness had been considered to be the most important predictor of reading (Paulesu et al., 2001) and spelling acquisition (Babayiğit and Stainthorp, 2007; Vaessen and Blomert, 2013). Recently, however, several researchers have questioned its status in transparent orthographies, in both normal acquisition of reading and spelling (Babayiğit and Stainthorp, 2007) on the one side, and in learning disorders (Wimmer and Schurz, 2010; Bigozzi et al., 2016) on the other one. A better understanding of the role of phonological awareness in reading and writing thus requires the assessment of phonological awareness before the onset of formal literacy, since conventional acquisition of reading and writing exerts an autoregressive effect on phonological awareness (Nikolopoulos et al., 2006).

Letter-sound matching skills are particularly important for reading fluency in beginner readers (Vaessen and Blomert, 2013), but fluency quickly reaches full development in transparent orthographies, which reduces the importance of letter-sound matching skills. In opaque (Caravolas et al., 2001) and transparent orthographies (Landerl and Wimmer, 2008; Torppa et al., 2013), instead, letter-sound matching skills remain associated to later spelling performances, although the effect-size of this association has been questioned, on the basis of the argument that knowing which letter belongs to which speech sound is not as important as using this knowledge efficiently and automatically (Vaessen and Blomert, 2013). Finally, in contrast with the clear association between RAN and reading disorders in transparent orthographies (Torppa et al., 2013), the theoretical link between RAN and spelling is also debated (Nikolopoulos et al., 2006; Babayiğit and Stainthorp, 2007; Torppa et al., 2013; Vaessen and Blomert, 2013).

Interest in the beginning stages of literacy development has focused attention on the very early invented spelling created by young children prior to formal reading and spelling instruction. Invented spellings, meant both as children’s early attempts at writing (Read, 1971) and as children’s early attempts at reading (Liberman, 1971), have been considered as a marker of children’s phonological awareness, and of their knowledge of the phonemic segments (sounds) represented by an alphabet. This assumed that since pre-reading children did not have a visual image of words fixed in their memory, when they sought to represent words they did so based on articulatory features.

Several authors have claimed that literacy outcomes are better predicted by an association between phonological awareness and letter knowledge, rather than by tasks tapping into oral phonological skills only (Ouellette and Sénéchal, 2008; Pinto et al., 2009; Wimmer and Schurz, 2010; Hulme and Snowling, 2013). Blaiklock (2004) contributed to the understanding of the combination of phonological-orthographic representations in kindergarten by demonstrating that the orthographic representations of words actually mediate the relationship between phonological awareness and literacy processes. Pinto et al. (2009) also suggested that children’s conceptual knowledge of the writing system captures this interplay between phonological and orthographic representations of the words, strongly predicting literacy acquisition.

Typically, conceptual knowledge of the writing system is assessed by an invented spelling task, in which the participant creates sound-signs that correspond to their level of knowledge of the writing system, from simple signs that discriminate writing from drawing, to an awareness that longer words require more signs than shorter words, to a 1:1 correspondence between sounds and signs in a word, although signs are not alphabetically correct. This early cognitive skill refers to phonological-orthographic connectivity and encompasses the systematic (even if not conventional) matching of sounds with written letters, and the productive component of writing, the ability to graphically build and develop a stable pattern of orthographic signs (even if unconventional and incorrect). In this sense, this factor takes into account the combined contribution of phonological awareness with other skills that are related to literacy acquisition and impaired in children with a reading disorder, that is grapho-motor skills (see Berninger et al., 2008), and visual attention (see Germano et al., 2014). Conceptual knowledge of the writing system includes child’s knowledge of the print conventions, of the names of letters, and of the letter sounds (Niessen et al., 2011).

Notwithstanding recent advances in research on conceptual knowledge of the writing system, its unique contribution to children’s acquisition of reading and spelling needs to be better understood (Niessen et al., 2011). Our research on emergent literacy predictors of reading and reading disorders (Bigozzi et al., 2016), and spelling (Pinto et al., 2009) in the Italian language has found that, when the conceptual knowledge of a writing system was included with phonological awareness among kindergarten predictors, the predictive power of phonological awareness disappeared, probably because its effect was absorbed by the conceptual knowledge of the writing system and integrated with orthographic knowledge. These results bring further evidence to Wimmer and Schurz’s (2010) hypothesis that reading disorders are better explained by an early deficit in orthographic-phonological connectivity. Conceptual knowledge of the writing system is also a better predictor of reading and reading disorders (Bigozzi et al., 2016), and spelling in writing (Pinto et al., 2009), than children’s textual competence. In an emergent literacy perspective, textual competence is an ability that is inter-related with other kindergarten competences, and is considered a developmental precursor to conventional forms of reading and writing (Lonigan et al., 2000). Thus, the ability to connect the phonological and orthographic representations of a word, (i.e., conceptual knowledge of the writing system) seems to be a more important cognitive skill for predicting reading and writing acquisition than the ability to get to grips with the individual units of meaning conveyed by the word and to form a network of relations between words that are in the text (i.e., textual competence).

### Aims of the Study

The aim of this study was to determine whether RSD and SD shared the same predictive pattern in kindergarten in terms of emergent literacy skills. In particular, (1) we focused on children’s conceptual knowledge of the writing system, and (2) we tested in a transparent writing system whether the conceptual knowledge of the writing system is an antecedent of RSD and SD children’s common impairment in spelling, similarly to what was found for reading acquisition and reading disorders (Bigozzi et al., 2016). We also studied the role of phonological awareness, because its predictive role for reading and spelling skills in transparent orthographies is debated.

The Italian language, which is a transparent writing system, allows to explore the relationship between emergent literacy and reading and spelling disorders, and fill the gap with our understanding of such a relationship in the context of opaque languages (e.g., English). In addition, the higher degree of transparency in the sign-sound correspondence in comparison with the sound-sign correspondence, allows one to clearly identify two clinical groups, RSD and SP, and run a comparative analysis between them and with the reference population.

The present study addressed these aims by carrying out a 4-year prospective cohort study. From a methodological perspective, a prospective cohort study shares the advantages of a longitudinal approach. However, previous longitudinal studies on reading and spelling disorders included only pupils from the population at risk of SD or RSD (e.g., familiarity or specific language impairment, see for instance Lyytinen et al., 2004), but excluded all those children with reading and/or spelling disorders that are present in the population not at risk. We designed a prospective cohort study so as to include all children from the natural population, at-risk and not-at-risk for learning disorders. From this general population, the SD and RSD samples were extracted from the same cohort, and were compared to the same control group. This approach provides a better control of potentially confounding variables (e.g., socio-economic status), and allows to better understand the relation between reading and spelling disorder. A prospective cohort study presents a further advantage. It allows to assess predictors of reading and spelling disorder symptoms manifesting in the third grade among children’s early skills in kindergarten, before the onset of formal literacy (i.e., before children’s early skills are influenced by the autoregressive effect of conventional learning of reading and spelling in primary school).

We expect the RSD and SD groups to show an impaired conceptual knowledge of the writing system in kindergarten, when compared to the control group (hypothesis 1). We expect the RSD and SD groups to show no impairment in phonological awareness or textual competence, when compared to the control group (hypothesis 2). Finally, we expect the SD and RSD groups to show no significant differences between each other in phonological awareness, conceptual knowledge of the writing system, and textual competence (hypothesis 3).

## Materials and Methods

### Participants

We followed a cohort of 642 Italian children from a mid-sized city in Central Italy (mean age: 4.98 ± 0.31 years; 299 girls and 343 boys) for 4 years, from the last year of kindergarten to the third grade. From this sample, we had previously excluded children showing a formal mastery of reading and writing during kindergarten. The parents of the participants gave informed consent for the participation of their children in the study. The measures were administered at a time agreed upon with the school and with due adherence to the requirements of privacy and informed consent required by the Italian law (Law Decree DL-196/2003). Regarding the ethical standards for research, the study referred to the last version of the Declaration of Helsinki (World Medical Association, 2013). The present study was approved by the Ethical Committee of the Department of Psychology at the University of Firenze, Italy. In the third grade, from the cohort of children, three groups were identified: 18 RSD pupils (12 boys and six girls), 13 SD pupils (nine boys and four girls), and 611 normally reading and -spelling pupils (322 boys and 289 girls). Interestingly, the two clinical samples respected the boy:girl ratio typically found in the literature for both reading and spelling disorder (Moll et al., 2014). Thus, the control group (children without a reading and/or spelling disorder) also presented a prevalence of boys over girls.

In the Italian educational system, children typically start kindergarten at the age of three, and finish it when they are five. Children then start primary school when they are 6 years old. Primary school lasts five grades. The school year begins in mid-September and ends in mid-June. All classes participating in the study (kindergarten and primary school) were part of the same school district therefore they shared some characteristics: similar educational and teaching practices and middle socio-economical level. Most importantly, in Italy the formal teaching of literacy begins in primary school, and follows a specific curriculum, as set down in national law. All the participating kindergartens were following the national guidelines issued by the Ministry of Education, which were valid at the time of the study. Since all emergent literacy skills are strongly dependent on family or kindergarten practices (Lonigan et al., 2000), we checked that no schools were following a specific program on formal literacy, and that no participant was already able to read and write in a conventional way at the time of the kindergarten assessment.

An important characteristic of Italian schools is low mobility: families tend to live in the same neighborhood over several generations. Children generally attend school in the same area. Therefore, in this study, subject attrition through the three stages was extremely low.

### Research Design

We present 4-year longitudinal data from a study of children from kindergarten to third grade. Children’s emergent literacy skills were assessed in kindergarten, at the beginning of the last school year. Four years later, when the participants were in third grade, we singled out the pupils who had received a diagnosis of reading and spelling disorder and the ones with a diagnosis of spelling disorder, and retrospectively analyzed their emergent literacy skills, comparing their performances to their normally reading and normally spelling peers. To ensure that all pupils had equal opportunity to be flagged as RSD or SD, we checked that none of the children included in the control group had received a diagnosis of a specific learning disorder.

The RSD and SD participants had received their diagnosis from the clinical units of the Italian National Health System, which follows the International Classification of Mental Disorders, ICD-10 (World Health Organization, 1992). The clinical units gave the researchers of this study access to each SD and RSD child’s protocol, in accordance with local privacy laws and standards.

#### Clinical Groups

In the following, we describe the criteria to be included in the SD or RSD group. Each SD and RSD child had displayed difficulties learning and using academic skills for at least six months, despite the provision of targeted interventions. SD displayed difficulties with written expression, with an impairment in written spelling, grammar, or punctuation, as assessed by the Battery for the Assessment of Developmental Reading and Spelling Disorders (Sartori et al., 2007). RSD displayed inaccurate and slow word reading, as assessed by MT Battery of Reading (Cornoldi and Colpo, 1998). With regard to cut-off scores, Moll et al. (2014) demonstrated that the association between RSD and SD depends on what thresholds we set to decide who to include in the two clinical groups, thus in this study, we adopted strict criteria to form the groups. In the RSD group, children had a reading accuracy and fluency score below the fifth percentile, as well as a written spelling performance score below the fifth percentile. In the SD group children’s writing accuracy was lower than the fifth percentile, whereas their reading performance was above the fifth percentile (see **Table 1**). RSD and SD children did not show any intellectual disability, as assessed by the Wechsler Intelligence Scale for Children-III (Wechlser, 2006), were not affected by uncorrected visual or auditory acuity, any mental or neurological disorder, psychosocial adversity, lack of proficiency in Italian or inadequate educational instruction. These aspects were assessed through the clinical synthesis of the individual’s history (developmental, medical, family, and educational), school reports, and psycho-educational assessment.

**Table 1:** Cut-off scores, number and proportion of children falling below the cut-offs, reading speed (syllable/seconds), reading accuracy (number of errors), and writing accuracy (number of errors) of control group, RSD, and SD children in third grade (mean, standard deviations, and range).

	Cut-off (fifth percentile)	*N* (%)	Control group	RSD	*SD*
Reading speed	1.18	18 (2.80)	3.5 ± 1.2 (1.55-5)	1.15 ± 0.50 (0.90-1.18)	3 1.1 ± (1.50-3.90)
Reading errors	13	18 (2.80)	4.9 ± 3.50 (0-6)	15 ± 4.3 (13-19)	5 ± 4.8 (1-6)
Writing errors	11	31 (4.83)	4.31 ± 3.50 (0-8)	14.50 ± 2.80 (13-18)	15.30 ± 3.50 (14-20)


#### Control Group

Children just failing to meet the cut-off points of pathological performance (e.g., a performance of seventh percentile) were kept in the control group as their reading and spelling was not impaired at a clinical level, and represent a sample from the reference population. In Italy, psychopathologies or disabilities are identified by the local health authorities at the parents’ request (Law 104/1992; Law 170/2010; Ministerial Decree July 12, 2011). After the diagnostic procedure ends, the local health authority gives the papers to the parents, who deliver them to the school, so that the procedures of school inclusion can be started (Decree of the President of the Council of Ministers 185/2006). Specific learning disabilities can be detected by teachers too, by notifying the child’s family so that they can proceed to start a diagnostic procedure with the local health authorities (Inter-Ministerial Ministry of Education, Universities and Research-Ministry of Health Decree; 17/4/2013)^1^. At the time of the study, control group children were not affected by any type of pathology, nor were they included in a diagnostic procedure, or identified by the teachers as children with special educational needs.

### Measures

Preschoolers were evaluated through tests measuring emergent literacy skills (phonological awareness, textual competence, and conceptual knowledge of the writing system). All the children’s products were coded by two independent judges. Agreement between the judges was between 88 and 99%; cases of disagreement were resolved through discussion. All the measures reported acceptable and good reliability scores.

#### Phonological Awareness

##### Identification and production of sound patterns (Dowker and Pinto, 1993)

The children were exposed to two verbal stimuli, one containing rhymes, and the other a series of alliterating words. The instruction was: “Now I am going to tell you a poem, which is a bit like a story but not quite. And I would like you to make one up too.” They were asked to produce a poem of their own, with the stimuli acting as examples. The order of the two stimuli was counterbalanced. Three scores were derived: rhythm (children’s ability to reproduce the prosody); rhyme (children’s ability to detect the rhymes within the stimulus); and alliteration (children’s ability to detect alliterations within the stimulus). The alpha coefficient for this instrument was 0.82. From this test, three measures were derived.

##### Identification and production of rhythm

The children’s ability to reproduce the prosody (rhythm) was scored as follows: 0 no rhythm produced, 1 one rhythm produced, 2 two or more rhythms produced. Pupils’ scores ranged from 0 to 2. Agreement between the judges was 94%.

##### Identification and production of rhyme

The children’s ability to detect the rhymes within the stimulus was scored as follows: 0 no rhymes produced, 1 one rhyme produced, 2 two or more rhymes produced. Pupils’ scores ranged from 0 to 2. Agreement between the judges was 97%. An example of a poem with rhyme detection from a kindergarten participant was:

mi piacciono le farfalle [I like butterflies]
azzurre, rosse, e gialle [blue, red, and yellow]

##### Identification and production of alliteration

The children’s ability to detect alliterations within the stimulus was scored as follows: 0 no alliterations produced, 1 one alliteration produced, 2 two or more alliterations produced. Pupils’ scores ranged from 0 to 2. Agreement between the judges was 98%. An example of a poem with alliteration detection from a kindergarten participant was:

scivolano gli sciatori sciando [the skiers slide while they’re skiing]

##### Identification of phonemes (Dowker and Pinto, 1993)

The children were asked to identify similar words among triplets of words, two of which had a phoneme in common. The alpha coefficient for this instrument was 0.79. Agreement between the judges was 93%; cases of disagreement were resolved through discussion. Children were exposed to nine three-word sets, and had to identify the two words with the initial phoneme in common. In three series they had to identify the initial phoneme (e.g., PALO – PESCA – NOTTE), in three series they had to identify the intermediate phoneme (e.g., AGO – UGO – EVA), and in three series they had to identify the final phoneme (e.g., BORSA – PRATO – TRENO). The following score was assigned: 0 if children correctly coded 0 to 2 triplets, 1 if children correctly coded 3 to 5 triplets, and 2 if children correctly coded 6 to 9 triplets. Pupils’ scores ranged from 0 to 2.

#### Conceptual Knowledge of a Writing System

##### Invented spelling (Pinto et al., 2009)

The scoring procedure we developed aimed to measure the extent to which an unconventional (e.g., incorrect) response made by a kindergarten child captured two main features of the written alphabetic language: the phonetic structure of the words (i.e., the number and the type of phonemes) that the child represented and the level of orthographic representation he/she adopted., and were sensitive enough to classify the lower level responses of kindergarten children. Children’s early written productions were analyzed in a quantitative and also qualitative manner using three categories, measuring the children’s knowledge of the sound-sign correspondence but also of the word boundaries, word morphology, directionality of print, number and shapes of letters required/allowed to compose a word. The children were asked to draw and write, from which three different scores were obtained. The alpha coefficient for this instrument was 0.92. Two independent raters coded the children’s products. The inter-rater reliability was 94%. Disagreements were resolved by discussion between the two raters.

##### Conceptual knowledge of orthographic notation

The children were asked to write down their name, the words they knew, and the word ‘mela’ (apple), for a minimum of two items. This score defined how similar children’s signs were to conventional letters. Scores were assigned as follows: 0 for drawings, 1 for scribbles, 2 for forms similar to letters, 3 for sequences of well-shaped letters.

##### Conceptual knowledge of the orthographic variation of sound quantity

Children were asked to write down two long words (one given by the experimenter, one of their choice), and two short words (one given by the experimenter, one of their choice), for a total of four items. This score defined whether the children were aware of the numeric correspondence between sounds and signs (one sign per sound). Scores were assigned as follows: 0 for drawings; 1 for performances based on a non-correspondence between signs and sounds (words of the same length, or the longer word written shorter than the short word); 2 for performances in which the difference in length is present and correct, without a 1:1 correspondence between signs and sounds; 3 for performances in which the difference in length is present and correct, with a 1:1 correspondence between signs and sounds.

##### Conceptual knowledge of the orthographic variation of phonemic units

The children were asked to write two pairs of words, each of which were formed by two words with the same first part and only the last letter different, for a total of two items This score defined whether the children were aware that words which sound similar are also written in a similar way, with small variations. Scores were assigned as follows: 0 for drawings, 1 for performances in which the two words were written, either identically, or completely differently; 2 for performances with a partial equivalence and a partial differentiation, where the two parts do not correspond to sound variations, however; 3 for performances with a partial equivalence and a partial differentiation, in which the two parts correspond perfectly to variations in sounds.

#### Textual Competence

##### Story production (Spinillo and Pinto, 1994)

The children were asked to tell a narrative. In the Italian school, kindergarten and primary school, this type of instruction refers to the production of fictional stories. All participants understood the instructions well and produced fictional stories. The story was recorded, transcribed, and analyzed by two independent judges on three parameters: structure, cohesion and coherence. The inter-rater reliability was 91%. Disagreements were resolved by discussion between the two raters. The alpha coefficient for this instrument was 0.91.

##### Structure

The story structure was coded by eight elements: (a) title, (b) conventional story opening, (c) characters, setting, (d) problem, (e) central event, (f) resolution, (g) conventional story closing. The system to attribute the structure scores was:

First level, non-story (one point): simple descriptions of actions without any characteristics of narrative style such as a conventional story opening or conclusion;
Second level, sketch story (two points): introduction of the setting and the main character, conventional story opening is often present, but both the problem and resolution are missing;
Third level, incomplete story (three points): elementary narrative structure, setting and characters are introduced, often with a conventional story opening and conclusion, but a central event is missing;
Fourth level, essential story (four points): non-essential structural elements, such as setting, are missing;
Fifth level, complete story (five points): all eight elements are included, with only the title considered optional.

##### Causal cohesion

To assess the causal cohesion in children’s stories, all the causal linguistic elements were identified (e.g., because, thus, so, and the like). On the basis of the quantity of causal cohesive elements used in the stories, balanced by the total number of words, three increasing levels of causal cohesion were identified: absent (zero points), low (one point), medium (two points), and high (three points).

##### Temporal cohesion

To assess the temporal cohesion in children’s stories, all the temporal linguistic elements were identified (e.g., once upon a time, then, because, after that, therefore, and the like). On the basis of the quantity of temporal cohesive elements used in the stories, balanced by the total number of words, three increasing levels of temporal cohesion were identified: absent (zero points), low (one point), medium (two points), and high (three points).

##### Coherence

To analyze coherence in the children’s narratives, the number of incoherencies were identified. On the basis of the number of incoherencies, balanced by the total number of sentences, three increasing levels of cohesion were identified: absent (zero points), low (one point), medium (two points), and high (three points).

### Data Analysis

Each variable’s extreme outliers were identified and eliminated by observing the relative box-plots. Through examination of the skewness and kurtosis of each dependent variable’s probability distribution, we verified that all variables were normally distributed. The statistical software R version 3.2.0 (R Core Team, 2015) was used to perform a linear mixed effects (LME) analysis of the relationship between group type (SD, RSD, or control group) and the notational knowledge of a writing system, phonological awareness and textual awareness. Separate LME models were run for each DV with the lmer function from the packages lme4 (Bates et al., 2014) and lmerTest (Kuznetsova et al., 2015). Model fitting was done by employing restricted maximum likelihood (REML). Compared to standard linear regression models, LME models are well suited for the analysis of unbalanced data sets (e.g., Sikorska et al., 2015). LME analysis decomposes model effects into the contribution of a fixed component (here the group) and a random component (here the class nested within the school nested within the school district). By including random-effect factors, the model can take the hierarchical structure linked to these factors into account.

Including a by-school within district and by-class within school within district random slope for the group led to an overparameterized model (correlation of –1.00 or 1.00 of the intercepts and slopes for the random effects), so we simplified the final models to include random intercepts for district, for school within district, and for class within school within district, and by- district random slopes for group. Collinearity was not an issue: all fixed-effect correlations (|r|) were less than 0.35.

The fixed effect estimates are provided by regression coefficients. To obtain an “effect size” of the group effect on notational knowledge, phonological awareness and textual awareness, we computed the LME standardized regression coefficients (β). When group membership is dummy coded with the control group as the baseline, a change in group membership results in a change of β standard deviations in the outcome. The standardized regression coefficients, therefore, provide a measure of effect size akin to Cohen’s *d* by taking the hierarchical nature of the data into account.

Visual inspection of residual plots did not reveal any obvious deviations from assumptions of homoscedasticity or normality. *p*-values were obtained using the pbkrtest in R (Halekoh and Højsgaard, 2014) for likelihood ratio test and parametric bootstrapping (with 10,000 resamples), and the multcomp package (Hothorn et al., 2008) with a Tukey correction for multiple comparisons.

## Results

### Descriptive Results

In **Table 2** pupils’ performances (SD, RSD, and control group) in kindergarten skills are reported.

**Table 2:** Descriptive statistics of kindergarten measures: mean and standard deviation (minimum; maximum).

Construct	Measure	Control group	RSD	*SD*
Phonological awareness	Rhythm	1.05 ± 0.76 (0;2)	1.38 ± 0.59 (0;2)	0.92 ± 0.76 (0;2)
	Rhyme	1.13 ± 0.80 (0;2)	1.52 ± 0.60 (0;2)	1.23 ± 0.73 (0;2)
	Alliteration	0.64 ± 0.75 (0;2)	0.90 ± 0.63 (0;2)	0.67 ± 0.78 (0;2)
	Phonemes	1.04 ± 0.76 (0;2)	1.19 ± 0.51 (0;2)	0.92 ± 0.64 (0;2)
Conceptual knowledge of a writing system	Notation	2.12 ± 0.65 (0;2.3)	1.50 ± 0.74 (0;3)	1.42 ± 0.73 (0;2.3)
	Sound quantity	1.54 ± 0.58 (0;2)	1.19 ± 0.66 (0;2)	1.17 ± 0.72 (0;2)
	Phonemic units	1.52 ± 0.91 (0;3)	1.14 ± 0.84 (0;3)	0.88 ± 0.43 (0;1.5)
Textual competence	Structure	2.04 ± 1.53 (0;5)	1.71 ± 1.23	1.67 ± 0.89 (0;3)
	Causal cohesion	0.76 ± 0.58 (0;3)	0.86 ± 0.36 (0;1)	1.17 ± 0.58 (0;2)
	Temporal cohesion	1.28 ± 0.95 (0;3)	1.05 ± 0.67 (0;3)	1.00 ± 0.43 (0;2)
	Coherence	1.14 ± 0.69 (0;2)	1.00 ± 0.55	1.17 ± 0.72 (0;2)


### Differences in Predictors between SD, RSD, and Control Group

After applying a Box–Cox transformation to correct for skewness, a principal component analysis (PCA) was performed on the centered and scaled variables describing the conceptual knowledge of a writing system, that is, orthographic notation (FNScr), phonemic units (FNSuSe), and sound quantity (FNVarNum). The first PC was used as an index of conceptual knowledge of the writing system (CKWS 72% explained variance). The correlations between CKWS and FNScr, FNSuSe, and FNVarNum were 0.86, 0.83, and 0.85, respectively. By using the same procedure, we created a phonological awareness index (PA). The correlations between PA (74% of explained variance) and the variables rhythm (CFRit), rhyme (CFRim), alliteration (CFAllPro), and phonemes (CFfon) were 0.92, 0.66, 0.94 and 0.89, respectively. Likewise, an index of textual competence (TC) was created. The correlations between TC (74% of explained variance) and the variables structure (StoStr), causal cohesion (StoCau), temporal cohesion (StoTem), and coherence (StoCoe) were 0.92, 0.66, 0.89, and 0.94, respectively. **Table 3** reports the correlations between the three principal components, conceptual knowledge of the writing system, phonological awareness, and textual competence.

**Table 3:** Correlations between the three principal components, conceptual knowledge of the writing system (CKWS), phonological awareness (PA), and textual competence (TC).

	CKWS	PA	TC
CKWS	1.00		
PA	0.26^∗^	1.00	
TC	0.23^∗^	0.28^∗^	1.00


*^∗^*p* < 0.0001.*

**FIGURE 1:**
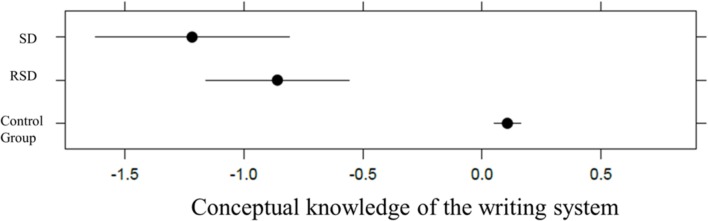
**Plot representation of SD, RSD, and control group in conceptual knowledge of the writing system in kindergarten**.

For conceptual knowledge of the writing system, including group in the model significantly increased the fit compared with a null, intercept-only model, χ_2ˆ2 = 7.93, *p* = 0.0189, *p*(bootstrap) = 0.0204, thus indicating a main effect of group. Tukey *post hoc* contrasts showed a statistically significant difference between the SD and control groups, *z* = 3.39, *p* = 0.0023, and between the RSD and control groups, *z* = 2.73, *p* = 0.0166, but not between the SD and RSD groups, *z* = 1.63, *p* = 0.2268. The β weights for the difference between the control group (baseline) and the RSD and SD groups were equal to –0.59 and –1.21, respectively. Conditional R_GLMMˆ2 (Johnson, 2014) was equal to 0.36 (variance explained by both fixed and random factors), with 12% of the explained variance due to the fixed-effects factor (see **Figure 1**).

**FIGURE 2:**
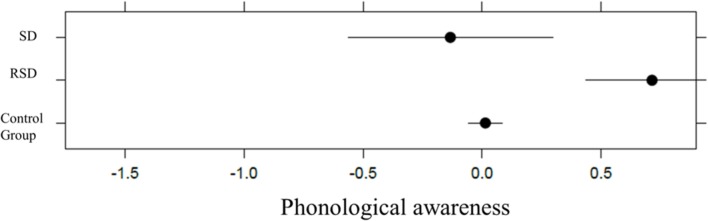
**Plot representation of SD, RSD, and control group in phonological awareness in kindergarten**.

For phonological awareness, we found no main effect of group, χ_2ˆ2 = 1.38, *p* = 0.5007, *p*(bootstrap) = 0.4510; Conditional R_GLMMˆ2 = 0.21, with 0.67% of the explained variance due to the fixed-effects factor (see **Figure 2**).

**FIGURE 3:**
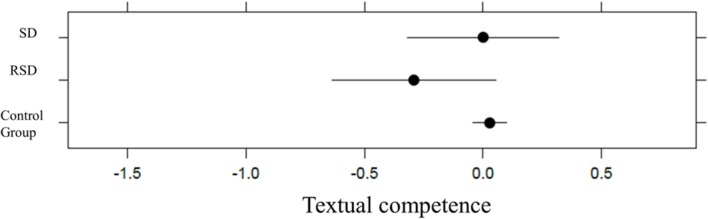
**Plot representation of SD, RSD, and control group in textual competence in kindergarten**.

Likewise, we found no main effect of group for textual competence, χ_2ˆ2 = 0.73, *p* = 0.6942, *p*(bootstrap) = 0.6471; Conditional R_GLMMˆ2 = 0.21, with 0.47% of the explained variance due to the fixed-effects factor (see **Figure 3**).

### Analyses Using Matched Control Group

In a different set of analyses, we only selected control participants from the classes where either an SD or an RSD child was found, to control for the effect of relevant confounding variables, i.e., socio-economic status, educational environment and gender. Two separate control groups were created: one for SD children (*n* = 62) and one for RSD children (*n* = 98). When only one SD or RSD child was present in a class, or when SD or RSD children present in a class had the same gender, controls were also matched for gender.

Linear mixed effects models were used to examine the group difference between SD or RSD children as measured by the conceptual knowledge of the writing system, phonological awareness, or textual competence dependent variables, with the same random-effect structure as described before. SD children had lower conceptual knowledge of the writing system scores than school-matched controls, χ_1ˆ2 = 10.37, *p* = 0.0019; the β weights for the difference between the control (baseline) and the SD and RSD groups were equal to –0.46 (*SE* = 0.22) and –0.87 (*SE* = 0.27), respectively. No statistically significant difference was found between SD children and controls with respect to phonological awareness, χ_1ˆ2 = 0.97, *p* = 0.3240, or textual competence, χ_1ˆ2 = 0.92, *p* = 0.3385.

Reading and spelling disorder children also showed lower conceptual knowledge of the writing system scores than school-matched controls, χ_1ˆ2 = 0.4.20, *p* = 0.0403; no statistically significant difference was found between RSD children and controls with respect to phonological awareness, χ_1ˆ2 = 0.12, *p* = 0.7272, or textual competence, χ_1ˆ2 = 0.49, *p* = 0.4824.

### Reading Performances in First Grade

To confirm the severity hypothesis, that specific spelling impairment might be a residual problem of pupils who have compensated earlier reading difficulties, we examined the reading performances of the three groups in first grade. According to the norms of the reading test used in this study (Cornoldi and Colpo, 1998), the cut-off score to diagnose an impairment in reading fluency is 0.51 syllables/second (fifth percentile). Control group pupils were reading 0.76 syllables/second (±0.12). RSD were already showing an impairment in reading in the first grade, as they were reading 0.40 syllables/second (±0.10). Instead, SD pupils just failed to meet the cut-off score of pathological performance (0.57 ± 0.18 syllables/second). In third grade, SD reading fluency performance improved drastically (see **Table 1**).

## Discussion

This 4-year study followed a cohort of Italian children from the last year of kindergarten to the third grade, when pupils were diagnosed with RSD or SD. Their kindergarten performance in conceptual knowledge of the writing system, their phonological awareness, and their textual competence were retrospectively compared to the performance of a control group peers. Our main findings are described below.

### RSD and SD Children versus NRS Peers

In kindergarten, SD and RSD children show an impaired conceptual knowledge of the writing system relative to control children without a reading and/or spelling disorder. The results from this cohort of children confirmed the results of a previous study on Italian children with a reading disorder (Bigozzi et al., 2016), and extend those finding to SD pupils too. In two previous studies (Pinto et al., 2009, 2012), conceptual knowledge of the writing system was shown to be an important predictor of spelling acquisition in first grade. This study extends the predictiveness of children’s invented spelling to the atypical learning trajectory of spelling too, as SD children were characterized by poor performances in this measure. Moreover, we found no evidence of differences in phonological awareness (in kindergarten) between SD, RSD, and control group children, thus supporting the idea that phonological awareness shows a limited power in predicting RSD (Wimmer and Schurz, 2010; Pinto et al., 2015; Bigozzi et al., 2016). Our results thus suggest that SD and RSD are associated disorders (Lyon et al., 2003; Bates et al., 2006; Egan and Tainturier, 2011).

That the conceptual knowledge of the writing system resulted to be the only statistically significant predictor does not show that phonological awareness is unrelated to the development of spelling skills (Babayiğit and Stainthorp, 2007; Vaessen and Blomert, 2013). Indeed, the conceptual knowledge of the writing system is a complex task, which integrates different cognitive, perceptual, and grapho-motor activities, with a phonological load (phonological coding of the input, identification of phonological units, ideation and choice of a transcoding system, and then execution of the transcoding system). Thus, we speculate that phonological awareness is integrated within conceptual knowledge of the writing system, rather than substituted by it, in agreement with previous theories stating that this factor is the medium through which phonological awareness exerts its effect on reading skills (Ouellette and Sénéchal, 2008). Given the multicomponential nature of conceptual knowledge of the writing system, besides the phonological load, other components could contribute to the predictivity of this factor on RSD and SD. For instance, the impairment could take place at the level of the visual-motor integration (Adi-Japha and Freeman, 2001). Future studies should explore these issues to increase our understanding of the specific contribution of conceptual knowledge of the writing system.

### RSD Children versus SD Peers

Our data show that SD and RSD children share a similar performance in phonological awareness and textual competence, and similar impairment in conceptual knowledge of the writing system. This result leaves still unanswered the question of whether the two clinical groups differ from each other in performances in kindergarten predictors. SD and RSD pupils do not show any difference in terms of performances in kindergartner skills.

We propose that RSD and SD children should be understood as belonging to two points on a continuum, rather than having two distinct pathologies. Although RSD and SD have similar levels of impairment in conceptual knowledge of the writing system, they show different spelling deficits, with a different level of severity: a spelling disorder (low severity) and reading and spelling disorder (high severity). We propose that this difference stems from the process of formal literacy. This proposal is consistent with the idea that variations in reading and spelling performances are influenced by many biological and contextual factors, (e.g., literacy environment at home and quality of instruction, see Hulme and Snowling, 2013).

The formalization and conventionalization that take place in primary school of the skills informally involved in the conceptual knowledge of the writing system in kindergarten requires pupils to perform two cognitive actions, spelling in writing and spelling in reading, with the former being more difficult than the latter (Newman et al., 1993; Wimmer and Schurz, 2010). Because of the asymmetry between the demands of spelling and reading in the formal setting, children diagnosed on the basis of a specific reading impairment, typically have writing problems too, while other pupils only have a significant impairment in writing. In this sense, we agree with Pennington’s (2006) severity hypothesis and Newman et al.’s (1993) residual problem hypothesis: the specific spelling impairment might be a residual problem of pupils who have managed to compensate for earlier mild reading difficulties. The analysis of participants’ reading performances in first grade supports this hypothesis, as SD pupils’ reading performances just failed to meet the cut-off score of pathological performance. However, the small sample sizes of the two clinical groups, SD and RSD, does not allow us to exclude the existence of significantly differing levels of impairment in conceptual knowledge of the writing system, which could also contribute to the potential explanation of the differential manifestation of the spelling deficit in SD and RSD. These considerations might apply specifically to transparent writing systems. If spelling and reading are asymmetric in all languages, such asymmetry is enhanced in transparent writing systems (Wimmer and Mayringer, 2002; Notarnicola et al., 2012). Indeed, opaque orthographies might induce higher rates of a combined reading and spelling disorder, whereas transparent orthographies could create the conditions for children to compensate their spelling difficulties when reading, especially by relying on the phonological route.

## Conclusion

The main conclusion of this study is that RSD and SD children in a transparent writing system share a common deficit among kindergartener’s skills: conceptual knowledge of the writing system. As Sampaio and Capellini (2014) highlighted, students who are exposed to literacy in a reflection-focused way show better literacy performances, as the orthographic processes become automatic and they can draw their attention to the content of the text, rather than to the correct spelling of it. Longitudinal studies on later reading and spelling performances may help identify early cognitive predictors, although it is important to note that such predictors do not determine disorders in an all or nothing way, as developmental interactions among early cognitive skills are likely and concur with the genetic risk of the manifestations of symptoms (Hulme and Snowling, 2013). At the practical level, identifying a plausible cognitive variable predicting later literacy disorders is critical for planning for educational intervention. Conceptual knowledge of the writing system could be a target skill to be included in screening tools for early identification of reading and spelling disorders. To this aim, future research should test its sensitivity (i.e., the proportion of true positives identified) and specificity (i.e., the proportion of true negatives identified), to validate invented spelling as a screening system (Andrade et al., 2015). An early intervention on skills that can potentially hinder the acquisition of reading and spelling can decrease the possibility of negative outcomes, also preventing a decrease in motivation and self-imposing restrictions on the literacy activities that children with a learning disorder often exhibit.

This study had several limitations. We found that phonological awareness is predictive of RSD when integrated with conceptual knowledge of the writing system. It would be interesting to consider different measures for phonological awareness, besides those used in the present study, as the impact of this construct on the prediction of reading and spelling disorders could depend on what component is measured (see Germano and Capellini, 2011). Although, we propose that reading and spelling disorders share a common core (conceptual knowledge of the writing system), other explanations may be possible, including impairment of other skills that are involved in the acquisition of orthographic knowledge (e.g., RAN, such as the sensitivity to orthographic regularities, letter knowledge and the implicit learning skills). Future studies should test the hypothesis that SD children are pupils who had previous reading difficulties and managed to resolve them. Moreover, future studies should also explore the factors contributing to help SD children to better cope with their reading difficulties.

## Author Contributions

All authors listed, have made substantial, direct and intellectual contribution to the work, and approved it for publication.

## Conflict of Interest Statement

The authors declare that the research was conducted in the absence of any commercial or financial relationships that could be construed as a potential conflict of interest.

## Footnotes

1 See https://www.european-agency.org/country-information/italy/national-overview/identification-of-special-educational-needs for more information on the identification of physical and mental disabilities and disorders in Italy.
